# Draft Genome Sequence of *Streptomyces* sp. JHA19, a Strain That Possesses β-d-Galactofuranosidase Activity

**DOI:** 10.1128/genomeA.01171-15

**Published:** 2015-10-08

**Authors:** Emiko Matsunaga, Yujiro Higuchi, Kazuki Mori, Kosuke Tashiro, Satoru Kuhara, Kaoru Takegawa

**Affiliations:** Department of Bioscience and Biotechnology, Faculty of Agriculture, Kyushu University, Fukuoka, Japan

## Abstract

By screening for microbes that exhibit β-d-galactofuranosidase (Gal*f*-ase) activity, a *Streptomyces* sp. strain, named JHA19, was isolated from a soil sample from Kagawa University, Japan, in 2010. Here, we report the results of whole-genome shotgun sequencing and found that the strain has four predicted Gal*f*-ase genes.

## GENOME ANNOUNCEMENT

*Streptomyces* species mostly inhabit soil and are Gram-positive bacteria that produce a variety of secondary metabolites, including antibiotics. Thus, *Streptomyces* spp. are valuable for the fermentation and pharmacological industries. In certain fungal species, β-d-galactofuranose (Gal*f*) is found as a component of polysaccharides and glycoconjugates (1–5). However, there have been no reports on the identification of genes encoding β-d-galactofuranosidase (Gal*f*-ases) that are involved in Gal*f* metabolism (6). Therefore, we screened soil samples for Gal*f*-ase activity and isolated a strain of *Streptomyces*, named JHA19, from soil at Kagawa University, Japan, in 2010. Here, we report the draft genome sequence of strain JHA19, which exhibited Gal*f*-ase activity.

This draft sequence was obtained by using a whole-genome shotgun sequencing strategy. Using GS FLX (Roche), 450-bp single-read sequencing was conducted. A total of 252 Mbp were generated from 6 × 10^5^ sequencing reads (32.7-fold coverage). Using the program Newbler version 2.7 to assemble the sequence data, 70 contigs (>100 bp) were generated. The largest contig length is 843,770 bp. Genome annotation was conducted with Glimmer version 3.02b and BLAST 2.2.26 to find genes and predict their functions, respectively. This draft genome of *Streptomyces* sp. strain JHA19 is 7.7 Mbp, with an average G+C content of 71.9%, and it contains 7,091 genes. The gene density is 1,086 bp/gene. The median and mean gene lengths are 825 bp and 948 bp, respectively.

Based on annotation of the whole-genome sequence of *Streptomyces* sp. JHA19, we found four predicted Gal*f*-ase genes, designated ORF0232, ORF1110, ORF2125, and ORF2812, located in contig0001, contig0002, contig0004, and contig0008, respectively (accession numbers BBRS01000001, BBRS01000002, BBRS01000004 and BBRS01000008, respectively). In the filamentous fungus *Aspergillus niger*, glycosyl hydrolase family 51 (GH51) and GH54 α-l-arabinofuranosidases (Ara*f*-ases), which catalyze the cleavage of α-l-arabinofuranose (Ara*f*), the structure of which resembles that of Gal*f*, exhibited both Gal*f*-ase and Ara*f*-ase activity (7). Domain prediction by the program Pfam (http://pfam.xfam.org/) revealed that all four proteins encoded by these open reading frames (ORFs) contain Ara*f*-ase-related domains. However, only the ORF1110 protein includes a GH2 domain, which has not been well characterized. In fact, we confirmed that proteins of ORF0232, ORF2125, and ORF2812 showed both Gal*f*-ase and Ara*f*-ase activities and that the ORF1110 protein exhibited Gal*f*-ase-specific activity (8).

### Nucleotide sequence accession numbers.

The contig sequences of *Streptomyces* sp. strain JHA19 have been deposited at DDBJ/EMBL/GenBank under the accession numbers BBRS01000001 to BBRS01000070.
